# The Dr. House Effect: Experts' Impoliteness Influences Persuasion

**DOI:** 10.1002/pchj.70081

**Published:** 2026-01-19

**Authors:** Teresa Garcia‐Marques, Rita Silva, Filipe Loureiro, Ana Lapa

**Affiliations:** ^1^ William James Center for Research ISPA‐Instituto Universitário Lisbon Portugal

**Keywords:** attitude change, Dr. House, expertise, persuasion, politeness

## Abstract

We examine whether polite or impolite speech affects experts' persuasiveness. Across three studies, experts using impolite language proved more persuasive than when polite (as portrayed by the Dr. House TV character) informing persuasion and politeness research.

## 1 Introduction

Dr. House, the ill‐tempered doctor from the TV series, exemplifies an expert who can convincingly persuade despite his impoliteness. Inspired by this character, we tested reactions to polite versus impolite expert and non‐expert communications (the “Dr. House‐effect”).

Persuasion research shows experts and likable sources are generally more persuasive than non‐experts and unlikable sources (e.g., Petty et al. 1998; see Pornpitakpan 2004). Yet, Ziegler and Diehl (2001) found that when both likability and expertise are high or low (i.e., a likable expert or an unlikable non‐expert), message scrutiny increases, and under strong arguments, a dislikable expert can outperform a likable one—an effect rarely examined since.

Communication style (e.g., enthusiastic, aggressive, polite) also shapes credibility. Aggressive language reduces scientists' credibility, and excessive enthusiasm can lower perceived integrity (König and Jucks 2019). Yuan et al. (2019) showed politeness may weaken expertise's impact on perceived message quality and likability—whereas politeness theory (Brown and Levinson 1987) assumes politeness matters more for non‐experts than experts. Thus, more studies are needed to examine how politeness affects persuasion when expertise varies.

We tested this across three experiments. Experiments 1 and 2 differed only in measurement order. Experiment 3 used an online sample and assessed additional perceptions. We hypothesized that impolite experts would be more persuasive than polite experts, while politeness would benefit non‐experts (the Dr. House‐effect; supplemental materials and results: https://osf.io/m2rhd/?view_only=8dd040ca8632437d88ca8413c186e0db).

## 2 Experiment 1 and 2

### 2.1 Participants and Design

Ninety‐six high school students (*M*_age_ = 16.30, SD = 1.20; 65 female) participated in Experiment 1, and ninety‐one (61.5% female; *M*_age_ = 17.51, SD = 0.81) in Experiment 2. Classes were randomly assigned to a 2 (expert vs. non‐expert) × 2 (polite vs. impolite) design. Sample sizes provided adequate power (*f* = 0.28, *α* = 0.05, power = 80).

### 2.2 Procedure

After consent, participants heard a message (female voice) emphasizing the importance of skincare. The message was framed and pre‐tested to be polite (e.g., “A beautiful skin makes us feel good about ourselves, and that makes us more beautiful”) or impolite (e.g., “Being ugly doesn't mean that you don't need to take care of your skin. You may not get any prettier, but your skin can”), delivered with a warm or cold (i.e., arrogant) vocal tone. The communicator was introduced as a dermatology specialist (expert) or an adolescent's mother (non‐expert).

In Experiment 1, participants rated (7‐point scales) topic personal relevance (e.g., *how frequently they thought about skincare*), skincare attitudes (e.g., *agreement with the message*; *intention to care for skin*), and source perceptions (e.g., *warmth*, *competence*). In Experiment 2, measurement order was reversed, and attitudes were assessed first with additional items (e.g., “The cleansing gel is an essential product”).

### 2.3 Results

#### 2.3.1 Manipulation Checks

Polite messages were rated more polite than impolite ones (Exp.1: *M* = 4.01, SD = 1.07 vs. *M* = 2.31, SD = 1.29, *F*(1, 92) = 53.90, *p* < 0.001, *η*_*p*_^2^ = 0.37; Exp.2: *M* = 5.00, SD = 1.41 vs. *M* = 3.82, SD = 1.42, *F*(1, 87) = 15.53, *p* < 0.001, *η*_*p*_^2^ = 0.15). Experts were judged more competent than non‐experts (Exp.1: *M* = 4.01, SD = 0.66 vs. *M* = 3.75, SD = 0.47, *F*(1, 92) = 4.02, *p* = 0.047, *η*_*p*_^2^ = 0.04; Exp.2: *M* = 4.47, SD = 1.28 vs. *M* = 3.71, SD = 1.21, *F*(1, 87) = 7.57, *p* = 0.007, *η*_*p*_^2^ = 0.08).

#### 2.3.2 Attitudes

**FIGURE 1:**
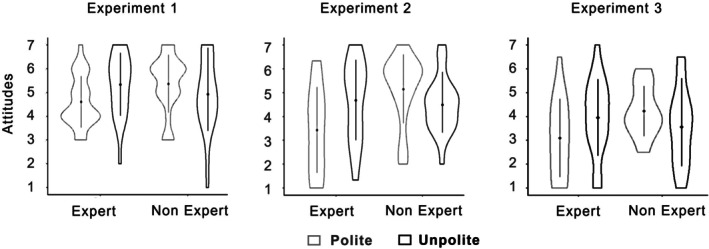
Joint effects of expertise and politeness on attitudes. Violin plots represent the distribution of attitude scores across the three experiments. The width of each violin reflects the density of responses, and the central dots indicate mean values. Across studies, impolite experts elicited more favorable attitudes than polite experts, whereas politeness increased persuasiveness for non‐experts, illustrating the Expertise × Politeness interaction.

Female participants reported more favorable attitudes than males (Exp.1: *M* = 5.20, SD = 1.19 vs. *M* = 4.68, SD = 1.54, *F*(1, 88) = 4.42, *p* = 0.038, *η*_*p*_^2^ = 0.04; Exp.2: *M* = 4.93, SD = 1.28 vs. *M* = 3.46, SD = 1.82, *F*(1, 83) = 15.39, *p* < 0.001, *η*_*p*_^2^ = 0.16), but gender did not interact with manipulations. Both studies revealed an expertise × politeness interaction (Exp.1: *F*(1, 88) = 4.67, *p* = 0.033, *η*_*p*_^2^ = 0.05; Exp.2: *F*(1, 83) = 4.28, *p* = 0.042, *η*_*p*_^2^ = 0.05). Impolite experts were more persuasive than polite experts, while politeness increased persuasion for non‐experts (see Figure 1). This pattern supports the Dr. House effect, indicating a detrimental effect of politeness when combined with expertise, in contrast to impoliteness (Exp1: *t*(88) = 2.14, *p* = 0.036, *d =* 0.45; Exp2: *t*(83) = 2.37, *p* = 0.026, *d* = 0.52).

#### 2.3.3 Additional Analysis

Topic relevance correlated with attitudes (Exp.1: *r* = 0.42; Exp.2: *r* = 0.62, *p*'s < 0.001). In a GLM, relevance showed a main effect (Exp.1: *F*(1, 88) = 14.91, *p* < 0.001, *η*_*p*_^2^ = 0.14, Exp.2: *F*(1, 70) = 7.06, *p* = 0.010, *η*_*p*_^2^ = 0.09), but no interaction (all *p*s > 0.25) with expertise politeness, nor a three‐way interaction. Importantly, relevance did not moderate the expertise × politeness interaction, which remained significant (Exp.1: *F*(1, 88) = 7.39, *p* = 0.008, *η*_*p*_^2^ = 0.08; Exp.2: *F*(1, 70) = 4.27, *p* = 0.042, *η*_*p*_^2^ = 0.06).

## 3 Experiment 3

### 3.1 Participants and Design

Seventy‐eight US students (51% female, *M*_age_ = 22.3, SD = 3.04) recruited on Prolific were randomly assigned to the same 2 × 2 design (two participants with participation times < 2.50 SD were excluded).

### 3.2 Procedure

An English translation and adaptation of the messages was presented in written form (gender‐neutralized) via Qualtrics. The procedure matched Experiment 2, with additional measures of perceived bias (e.g., *How much would you see the person as having a biased perspective*?; see Wallace et al. 2020), trustworthiness (e.g., *How much would you see the person as trustworthy*?; see Ziegler and Diehl 2001), and persuasive intent (e.g., *To persuade me to buy this type of product*).

### 3.3 Results

#### 3.3.1 Manipulation Checks

Polite versions were rated more polite (*M* = 4.82, SD = 1.02 vs. *M* = 3.35, SD = 1.44, *F*(1, 74) = 27.41, *p* < 0.001, *η*_*p*_^2^ = 0.27) and experts as more competent (*M* = 4.85, SD = 1.17 vs. *M* = 4.05, SD = 1.58, *F*(1, 74) = 6.79, *p* = 0.011, *η*_*p*_^2^ = 0.08).

#### 3.3.2 Attitudes

Replicating Experiments 1 and 2, an expertise × politeness interaction emerged, *F*(1, 704) = 5.48, *p* = 0.022, *η*_*p*_^2^ = 0.07. Impolite experts were more persuasive than polite experts (see Figure 1), *t*(74) = 1.78, *p* = 0.040, *d* = 0.41, whereas no difference emerged for non‐experts, *t*(74) = −1.37, *p* = 0.175. No other effects were significant (all *p*s > 0.25).

The effect remained significant when engagement was included in GLM, *F*(1, 70) = 6.15, *p* = 0.016, *η*_*p*_^2^ = 0.08, with no three‐way interaction, *F* = 0.27. Engagement interacted only with politeness, *F*(1, 70) = 4.41, *p* = 0.039, *η*_*p*_^2^ = 0.06: politeness reduced persuasion among highly engaged participants (*β* = −0.55, *t*(70) = 1.70, *p* = 0.093) but increased it among less engaged ones (*β* = 0.41, *t*(70) = 1.30, *p* = 0.196). No other effects were significant.

#### 3.3.3 Perceived Persuasive Intent

Polite messages were judged to have stronger persuasive intent (*M* = 5.65, SD = 1.12) than impolite ones (*M* = 4.88, SD = 1.46), *F*(1, 74) = 5.34, *p* = 0.024, *η*_*p*_^2^ = 0.07. Both expertise and the expertise × politeness interaction were nonsignificant (*p*s > 0.25). Controlling for intent did not affect the Dr. House effect. Additionally, the GLM revealed null effects of this variable (all *p* > 0.21).

#### 3.3.4 Perceived Trustworthiness and Bias

Only an expertise × politeness interaction emerged, *F*(1, 74) = 5.36, *p* = 0.023, *η*_*p*_^2^ = 0.07, indicating that, for non‐experts, polite messages were judged less biased than impolite messages (*M* = 2.08, SD = 0.37 vs. *M* = 3.55, SD = 0.35, *t*(74) = 2.53, *p* = 0.013, *d* = 0.59), whereas experts' bias perceptions did not differ (*t*(74) = 0.73). All other effects were nonsignificant (all *p*s > 0.201).

## 4 Discussion

Across three experiments, experts were more persuasive when impolite, a phenomenon we term the Dr. House‐effect. Politeness mainly boosted non‐experts' but not experts' credibility.

The effect proved robust across topic relevance and perceived persuasive intent, suggesting it does not stem from elaboration or resistance processes (Petty et al. 1998). Future research should test these mechanisms directly and whether it reflects expectancy violations (e.g., Burgoon et al. 2002) or other credibility‐related processes (e.g., trustworthiness, bias perceptions). In Experiments 1 and 2, class‐level randomization may have introduced confounds, but overall, that did not impact the consistent detection of the effect. Further investigation should address the levels of the source's perceived politeness and expertise (higher in Experiment 1 than 2), gender matching, and relevant message dimensions (e.g., familiarity, surprise) to rule out alternative explanations. Exploring other communication styles and cues beyond likability will clarify the generalization of the effect.

## Funding

Fundação para a Ciência e Tecnologia, I.P., in the context of the project I&D: UID/04810/2025—WJCR.

## Ethics Statement

Studies complied with WJCR standards and the Helsinki Declaration. Data/materials are available at https://osf.io/m2rhd/?view_only=8dd040ca8632437d88ca8413c186e0db.

## Consent

Was obtained from schools, parents, and participants.

## Conflicts of Interest

The authors declare no conflicts of interest.

## Supporting information


**Data S1:** Supporting Information.

## References

[pchj70081-bib-0001] Brown, P. , and S. C. Levinson . 1987. Politeness: Some Universals in Language Usage. Vol. 4. Cambridge University Press.

[pchj70081-bib-0002] Burgoon, J. K. , J. A. Bonito , A. Ramirez Jr. , N. E. Dunbar , K. Kam , and J. Fischer . 2002. “Testing the Interactivity Principle: Effects of Mediation, Propinquity, and Verbal and Nonverbal Modalities in Interpersonal Interaction.” Journal of Communication 52, no. 3: 657–677. 10.1111/j.1460-2466.2002.tb02567.x.

[pchj70081-bib-0003] König, L. , and R. Jucks . 2019. “Influence of Enthusiastic Language on the Credibility of Health Information and the Trustworthiness of Science Communicators: Insights From a Between‐Subject Web‐Based Experiment.” Interactive Journal of Medical Research 8, no. 3: e13619. 10.2196/13619. 31411138 PMC6711041

[pchj70081-bib-0004] Petty, R. E. , D. T. Wegener , and P. H. White . 1998. “Flexible Correction Processes in Social Judgment: Implications for Persuasion.” Social Cognition 16, no. 1: 93–113. 10.1521/soco.1998.16.1.93.

[pchj70081-bib-0005] Pornpitakpan, C. 2004. “The Persuasiveness of Source Credibility: A Critical Review of Five Decades' Evidence.” Journal of Applied Social Psychology 34, no. 2: 243–281. 10.1111/j.1559-1816.2004.tb02547.x.

[pchj70081-bib-0006] Wallace, L. E. , D. T. Wegener , and R. E. Petty . 2020. “When Sources Honestly Provide Their Biased Opinion: Bias is a Distinct Source Perception With Independent Effects on Credibility and Persuasion.” Personality and Social Psychology Bulletin 46, no. 3: 439–453. 10.1177/01461672198586. 31282841

[pchj70081-bib-0007] Yuan, S. , A. Dudo , and J. C. Besley . 2019. “Scientific Societies' Support for Public Engagement: An Interview Study.” International Journal of Science Education, Part B 9, no. 2: 140–153. 10.1080/21548455.2019.1576240.

[pchj70081-bib-0008] Ziegler, R. , and M. Diehl . 2001. “The Effect of Multiple Source Information on Message Scrutiny: The Case of Source Expertise and Likability.” Swiss Journal of Psychology 60, no. 4: 253. 10.1024/1421-0185.60.4.253.

