# Supplementary material for: The Dr. House Effect: Experts' Impoliteness Influences Persuasion
Source: Psych J. 2026 Jan 19;15(1):e70081. doi: 10.1002/pchj.70081 (PMC12815492; doi:10.1002/pchj.70081)
Supplement: Supplementary file 1 — Data S1: Supporting Information. [file PCHJ-15-e70081-s001.docx]

**Supplemental Materials**

**Experiment 1**

**Method**

***Materials***

The message employed in this study was developed with the aim of persuading participants to take care of their skin, particularly their facial skin, by maintaining its cleanliness and hydration. The expertise of the source was manipulated by informing participants that they would listen to a message delivered by an esthetician with expertise in clinical studies in dermatology. Alternatively, participants were informed that the message was presented by a regular citizen, specifically, a mother of an adolescent.

The politeness of the source was manipulated by introducing additional comments alongside the message content and making changes in the tone of voice used in the recorded tape. The voice was either sweet and tender or arrogant and cold, emulating the character of Dr. House. For the message presented by an impolite source, comments such as *"Being ugly doesn’t mean that you don't need to take care of your skin. You may not get any prettier, but your skin can"* and *"It’s easy, so give it a try: put on some makeup, hide all your imperfections, and fool yourself into thinking it actually works"* were included. In contrast, for the message delivered by a polite source, comments like *"A beautiful skin makes us feel good about ourselves, and that makes us more beautiful"* and *"Don't hide your imperfections with makeup; instead, take care of them and prevent them from appearing by taking care of your skin"* were added.

These messages were pre-tested with separate groups of 15 individuals who evaluated the two sources in a random order using three seven-point semantic differentials: pleasant vs. unpleasant, warm vs. cold, and polite vs. impolite. These three evaluations were averaged for the two sources, and significant differences between the likeable (*M* = 4.93, *SD* = 1.48) and unlikeable source (*M* = 3.53, *SD* = 1.36) were observed, *t*(14) = 3.31, *p* = .005, *d* = 0.85, confirming the adequacy of the messages.

***Procedure***

A total of six high-school classes were invited to take part in a study about skincare during adolescence. Each class was randomly divided into two groups. Classes were randomly assigned to listen to a recorded message delivered by an unlikeable source, while the other half received a message from a likeable source.

After obtaining informed consent, participants were informed that they would listen to a message about skincare and were provided with a booklet containing instructions. Inside the booklet, half of the participants in each group were informed that the message would be presented by an aesthetician with expertise in clinical studies in dermatology, while the other half were told that the message was from a mother of an adolescent. Participants were instructed to attentively listen to the message and then refer to the booklet to respond to a series of questions related to the topic.

The message was presented to participants in a recorded format, and immediately after listening to it, they used a seven-point scale to select a number that best represented their opinions. The first three questions aimed to assess the relevance of the skincare issue for each participant (i.e., how relevant the topic was to them; their level of preexisting knowledge about skincare; and how frequently they thought about skincare). The fourth and fifth questions assessed their attitudes towards the position presented in the message (i.e., how much they agreed with the position defended in the message; and how much they believed they should take care of their skin). Subsequently, participants were asked to characterize the source of the message using seven-point scales (pleasantness-unpleasantness, warmth-coldness, expertise-non-expertise on the topic, competence-incompetence).

**Results**

***Manipulations checks***

Ratings of the politeness-related items (i.e., warmth and likability) were significantly correlated (*r*=.85, *p*<.001) and were averaged to create a measure of perceived likability and analysed in an ANOVA. The manipulation of politeness proved effective, as polite communications received higher evaluations of politeness (*M*=4.01, *SD*=1.07 vs. *M*=2.31, *SD*=1.29; *F*(1, 92)=53.90, *p*<.001, η_p_²= .37). No other effects reached significance (Expertise main effect, *F*= 0.46; interaction, *F*= 0.47).

The two items measuring perceived expertise (i.e., expertise and competence) were significantly associated (*r*=.47, *p*<.001), and their averages were analysed within the design. The manipulation of expertise was effective, with experts receiving higher evaluations of expertise than non-experts (*M*=4.01, *SD*=0.66 vs. *M*=3.75, *SD*=0.47; *F*(1, 92)=4.02, *p*=.047, η_p_²=.04. The polite communicator was perceived as more expert (*M*=4.06, *SD*=0.49) than the impolite communicator (*M*=3.69, *SD*=0.60; *F*(1, 92)=8.65, *p*=.004, η_p_²=.09), but no interaction occurred (*F*=0.75).

***Attitudes***

Female participants reported more favorable attitudes than males (*M=*5.20, *SD=*1.19 vs. *M=*4.68, *SD=*1.54, *F*(1, 88)=4.42, *p*=.038, *η_p_²=*.04), but gender did not interact with manipulations. There was an expertise x politeness interaction, *F*(1, 88)=4.67, *p*=.033, *η_p_²=*.05. Impolite experts were more persuasive than polite experts while politeness increased persuasion for non-experts (see Figure 1). This pattern supports the Dr. House effect, indicating a detrimental effect of politeness when combined with expertise, in contrast to impoliteness, *t*(88)= 2.14, *p*=.036, *d=* 0.45.

**Figure 1**

*Joint effects of Expertise and Politeness on attitudes*


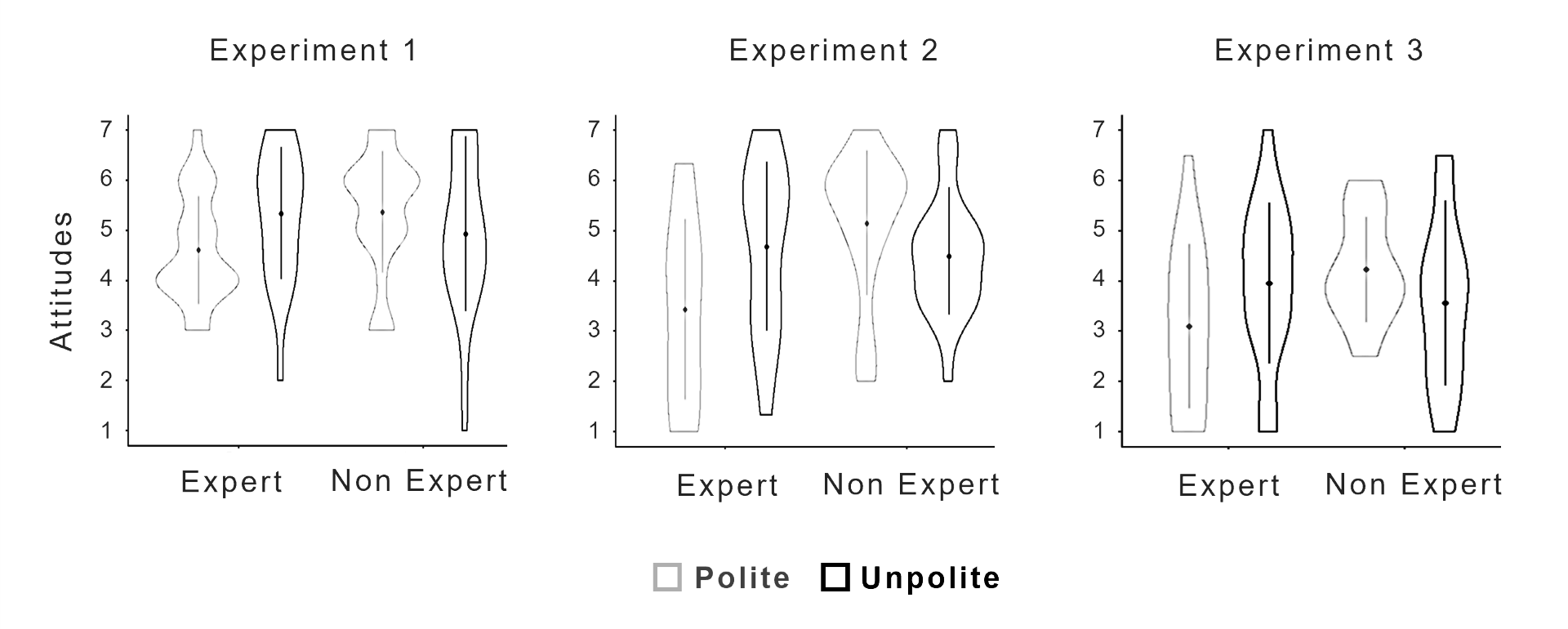


***Additional analysis***

Topic relevance correlated with attitudes (*r*=.42, *p*<.001). In a GLM, relevance showed a main effect, *F*(1, 88)=14.91, *p<*.001, *η_p_²=*.14, but no interaction with expertise, *F*=0.12, politeness, *F*(1, 88)=1.33, *p*=.252, *η_p_²*=0.01, nor a three-way interaction, *F=*0.27. Importantly, relevance did not moderate the expertise x politeness interaction, which remained significant, *F*(1, 88)=7.39, *p=*.008, *η_p_²=*.08.

**Experiment 2**

**Method**

***Materials***

Materials were the same as in Experiment 1.

***Procedure***

Procedure was the same as in Experiment 1, with the exception that, in Experiment 2, participants first reported their attitudes (i.e., The face cleansing gel is an essential product; True facial cleansing is done with cleansing gel; I take better care of my facial skin when I use cleansing gel; I would feel my skin cleaner if I used cleansing gel), the message source (pleasant-unpleasant; warm-cold; polite-impolite; expert-non expert; competent-incompetent), and the topic’s relevance (In my opinion, beauty is very important; It is extremely important to have beautiful skin; The topic of cosmetics is of little relevance).

**Results**

***Manipulations checks***

Ratings of the politeness-related items (i.e., warmth and likability) were significantly correlated (*r*=.88, *p*<.001) and were averaged to create a measure of perceived likability and analyzed in an ANOVA. The manipulation of politeness proved effective, as polite communications received higher evaluations of politeness (*M*=5.00, *SD*=1.41 vs. *M*=3.82, *SD*=1.42; *F*(1,87)=15.53, *p*<.001, η_p_²=.15). No other effects reached significance (Expertise, *F*=0.67; interaction, *F*=0.12).

The two items measuring perceived expertise (i.e., expertise and competence) were significantly associated (*r*=.58, *p*<.001), and their averages were analyzed within the design. The manipulation of expertise was effective, with experts receiving higher evaluations of expertise than non-experts (*M*=4.47, *SD*=1.28 vs. *M*=3.71, *SD*=1.21; *F*(1,87)=7.57, *p*=.007, η_p_²=.08. The interaction was marginally significant, *F*(1,87)=3.28, *p*=.074, η_p_²=.04, suggesting that the manipulation was more effective for the impolite source (*M*_Expert_=4.56, *SD*=1.12; *M*_Non-expert_=3.37, *SD*=1.08) than for the polite source (*M*_Expert_=4.38, *SD*=1.44; *M*_Non-expert_=4.14, *SD*=1.26).

***Attitudes***

Female participants reported more favorable attitudes than males (*M=*4.93, *SD=*1.28 vs. *M=*3.46, *SD=*1.82, *F*(1, 83)=15.39, *p*<.001, *η_p_²=*.16), but gender did not interact with manipulations. There was an expertise x politeness interaction, *F*(1, 83)=4.28, *p*=.042, *η_p_²=*.05. Impolite experts were more persuasive than polite experts while politeness increased persuasion for non-experts (see Figure 1). This pattern supports the Dr. House effect, indicating a detrimental effect of politeness when combined with expertise, in contrast to impoliteness, *t*(83)=2.37, *p*= .026, *d=*0.52).

***Additional analysis***

Topic relevance correlated with attitudes (*r*=.62, *p*<.001). In a GLM, relevance showed a main effect, *F*(1, 70)=7.06, *p*=.010, *η_p_²*=.09, but no interaction with expertise, *F*(1, 70)=2.37, *p*=.128, *η_p_²*=.03, politeness, *F*(1, 70)=0.75, nor a three-way interaction, *F*(1, 70)=0.01. Importantly, relevance did not moderate the expertise x politeness interaction, which remained significant, *F*(1, 70)=4.27, *p*=.042 *η_p_²*=.06.

**Experiment 3**

**Method**

***Materials and procedure***

A translation and adaptation of the message was presented in written form to neutralize the source's gender effects. The study was conducted through a Qualtrics online survey, closely following Experiment 2’s procedures. In addition to assessing topic relevance, it evaluated perceived bias (How much would you see the person as having a biased perspective? How much would you see their opinion as a product of personal bias? 1-very biased, 7-very unbiased; see Wallace et al., 2020), trustworthiness (How much would you see the person as trustworthy? How much would you perceive them as a trustworthy person? 1-very untrustworthy, 7-very trustworthy; see Ziegler & Diehl, 2001), and perceived persuasive intent (agreement with: To persuade me to buy this type of product; To shape my opinion towards the product; To manipulate my opinion).

**Results**

***Manipulations checks***

The perceived politeness and expertise’s items compounded into two factors (the first explaining 79.9% of shared variance; Cronbach’s alpha = .85; and the second explaining 85.5%; Cronbach’s alpha = .91), being averaged into two indexes.

The polite message’s source received higher likability ratings (*M*=4.82, *SD*=1.02 vs. *M*=3.35, *SD*=1.44), F(1, 74)=27.41, *p*<.001, η_p_²=.27). Expertise showed no impact on likability perception (no main effect, *F*(1, 74)=1.88, *p*=.174, η_p_²=.02, and no interaction, *F*= 0.77).

The expert source was perceived as more expert (*M*=4.85, *SD*=1.17 vs. *M*=4.05, *SD*=1.58, *F*(1, 74)=6.79, *p*=.011, η_p_²=.08). Additionally, the polite message was associated with higher perceived expertise (*M*=4.82, *SD*=1.29 vs. *M*=4.10, *SD*=1.50, *F*(1, 74)=5.67, *p*=.020, η_p_²=.07). Their interaction was non-significant, *F*(1, 74)=1.79, *p*=.185, η_p_²=.03.

***Attitudes***

The averaged index of attitude items (explaining 85.2% of the variance; Cronbach’s alpha of .94) was analyzed within an ANOVA, revealing only the expected expertise x politeness interaction, *F*(1, 704)=5.48, *p*=.022, η_p_²=.07 (Main effect of expertise: *F*(1, 70)=1.31, *p*=.255, η_p_²= .02; Main effect of politeness: F= 0.26). Additionally, no significant effects of gender were observed (Main effect: *F*(1, 70)=3.54, *p*=.064, η_p_²=.05; Gender x politeness, *F*= 0.90; Gender x Expertise, *F*= 0.33; three-way interaction, *F*= 0.01). Simple analysis showed that, for an expert, being impolite promoted more favorable attitudes compared to being polite, *t*(74)=1.78, *p*=.040, *d*=0.41, while this was not observed for non-experts, *t*(74)=-1.37, *p*=.175.

Exploring the role of engagement, results of the General Linear Model revealed that the Dr. House effect, *F*(1, 70)=6.15, *p*=.016, η_p_²=.08, was not moderated by engagement (three-way interaction, *F*=0.27). Engagement only interacted with politeness, *F*(1, 70)=4.41, *p*=.039, η_p_²=.06 (main effect, *F*=0.28; engagement x expertise, *F*=0.72), moderating its effects: politeness negatively influenced attitude for highly engaged participants (*β*=-0.55, *t*(70)=1.70, *p*=.093) while favoring attitudes for those with low engagement (*β*=0.41, *t*(70)=1.30, p=.196).

***Perceived persuasive-intent***

Only politeness influenced persuasive-intent of the source: the polite message (*M*=5.65, *SD*=1.12) was perceived as having more persuasive-intent than the impolite message (*M*=4.88, SD=1.46), *F*(1, 74)=5.34, *p*=.024, η_p_²=.07 (main effect of expertise: *F*=0.01; interaction: *F*(1, 74)=1.48, *p*=.227, η_p_²=.02). Controlling for intent did not affect the Dr. House effect. Additionally, the General Linear Model revealed null effects of this variable (persuasive intent x expertise, *F*= 0.65; persuasive intent x politeness, *F*= 0.01; three-way interaction, *F*=0.67; main effect, *F*(1, 70)=1.05, *p*=.218, η_p_²= .01).

**Perceived trustworthiness and bias**

Our manipulations did not impact perceived trustworthiness (politeness: *F*(1, 74)=2.13, *p*=.148, η_p_²= .32; expertise: *F*(1, 74)=2.51, *p*=.118, η_p_²=.03; interaction: *F*(1, 74)=1.67, *p*=.201, η_p_²=.02) or perceived bias (politeness: *F*(1, 74)=1.61, *p*=.208, η_p_²=.02; expertise: *F*=0.00). The expertise x politeness interaction, *F*(1, 74)=5.36, *p*=.023, η_p_²=.07, indicated that, for experts, politeness had no effect on perceived bias, *t*(74)=0.73 (*M*_Polite_=3.26, *SD*=0.38; *M*_Impolite_=2.65, *SD*=0.35). Conversely, for non-experts, a polite message was perceived as less biased (*M*=2.08, *SD*=0.37) than an impolite one (*M*=3.55, *SD*=0.35), *t*(74)=2.53, *p*=.013, *d*=0.59).
